# First record of *Closterocerus chamaeleon*, parasitoid of the Eucalyptus Gall Wasp *Ophelimus maskelli* (Hymenoptera, Chalcidoidea, Eulophidae), in the New World

**DOI:** 10.3897/zookeys.504.9728

**Published:** 2015-05-19

**Authors:** Roger A. Burks, Jason L. Mottern, Nicole G. Pownall, Rebeccah Waterworth, Timothy D. Paine

**Affiliations:** 1 Entomology Department, University of California, Riverside, Riverside, CA, USA 92521

**Keywords:** Natural enemy, exotic pest, biological control

## Abstract

The uniparental parasitoid *Closterocerus chamaeleon* (Girault) is discovered to be fortuitously present on a population of the invasive Eucalyptus Gall Wasp *Ophelimus maskelli* (Ashmead) in Riverside, California. This is the first report from the New World of *Closterocerus chamaeleon*, which has proven to be a highly effective natural enemy of *Ophelimus maskelli* in the Mediterranean Basin. The taxonomy and identification of *Closterocerus chamaeleon* is discussed.

## Introduction

*Ophelimus maskelli* (Ashmead) (Hymenoptera: Eulophidae) is a uniparental pest, originally from Australia, which forms leaf galls on *Eucalyptus* in the Exsertaria, Latoangulata, and Maidenaria sections, causing premature leaf drop. When uncontrolled, it reached high enough populations in the Mediterranean Basin to become a major nuisance in addition to the damage inflicted on *Eucalyptus* (Protasov et al. 2007a). It was recently found in multiple localities in southern California (Burks et al. 2015), and efforts have since been underway to explore biological control possibilities.

*Closterocerus chamaeleon* (Girault) (Hymenoptera: Eulophidae), also originally from Australia, has been the most effective natural enemy of *Ophelimus maskelli* released in the Mediterranean Basin, showing strong potential for spreading to populations of the pest in distant locations, and proving able to successfully attack overwintering hosts (Laudonia et al. 2006; Rizzo et al. 2006; Mendel et al. 2007; Protasov et al. 2007b; Caleca 2010; Caleca et al. 2011).

Both *Ophelimus maskelli* and *Closterocerus chamaeleon* are in the family Eulophidae, but are distantly related, in the subfamilies Opheliminae and Entedoninae, respectively. Opheliminae is composed entirely of gall makers and associates, while Entedoninae contains parasitoids of a wide variety of arthropods (Bouček 1988).

**Figures 1–2.**
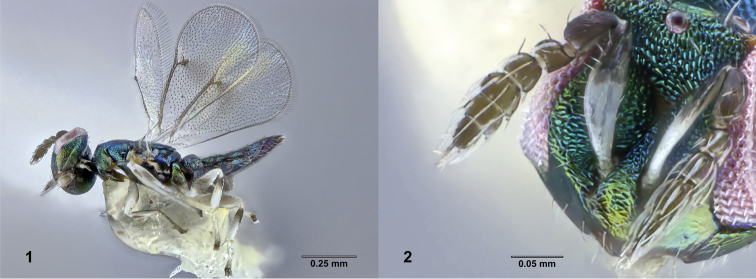
**1** *Closterocerus chamaeleon* reared from *Ophelimus maskelli* gall collected on UCR campus; body, lateral view. UCRCENT00412686 **2** Antennae and head of the same specimen, oblique anteromedial view.

The morphology of *Closterocerus chamaeleon* was recently reviewed by Protasov et al. (2007b), who also discussed the recent taxonomic history of the genus *Closterocerus* Westwood. Since then, Burks et al. (2011) discovered that 28S D2 and COI DNA data supported *Closterocerus* as distinct from the morphologically similar genera *Chrysonotomyia* Ashmead and *Neochrysocharis* Kurdjumov. Placement of *Closterocerus chamaeleon* in *Closterocerus* is based on the strongly curved transepimeral sulcus and the presence of a bare area on the fore wing anterior to the uncus, which are reasonably reliable features of the genus (Hansson 1990, 1994). The number of spines on the volsellar digitus of the male genitalia has more recently been used to distinguish *Chrysonotomyia* (Hansson 2004) from all similar genera, but males of *Closterocerus chamaeleon* are unknown. The morphological features separating *Closterocerus chamaeleon* (Fig. 1) from species in all of these genera remain as initially reported (Protasov et al. 2007b: figs 1–12): antennal scape brown in apical third and with ventral margin convex, F3 (1st funicular) shorter than next flagellomere and strongly narrowed asymmetrically basally (Fig. 2), mesosoma dorsally with uniformly reticulate surface sculpture, fore wing with faint infuscation near stigmal vein, legs white except usually with brown areas on femora and laterally on metatibia, and gaster with first tergite smooth and all others reticulate.

## Methods

*Eucalyptus* leaves with *Ophelimus maskelli* galls were placed in sealed containers awaiting emergence. A small amount of honey was placed on the inside of each container using a minutien pin probe. Photographs were taken using a Leica Imaging System with a Z16 APO A microscope, and stacked using Zerene Stacker (version 1.04). Terminology follows that of Gibson (1997).

## Results and discussion

Several individuals of *Closterocerus chamaeleon* were reared from overwintering galls of *Ophelimus maskelli* on *Eucalyptus* leaves collected from the University of California, Riverside (UCR) campus on March 20, 2015. This suggests that *Closterocerus chamaeleon* was also present in the area in 2014. This is the first report of *Closterocerus chamaeleon* from the New World. While we have found *Ophelimus maskelli* in multiple locations in Orange, Riverside, and San Diego counties in California (Burks et al. 2015), we have found *Closterocerus chamaeleon* only in Riverside. No individuals of *Closterocerus chamaeleon* have been intentionally imported or released in California, and therefore it was most likely accidentally introduced through the same avenue that established *Ophelimus maskelli* in the area. This is therefore a case of fortuitous accidental introduction of a beneficial parasitoid.

Some native Californian species of *Neochrysocharis* are similar to *Closterocerus chamaeleon*, but they are parasitoids of leaf-miners and differ from *Closterocerus chamaeleon* in one or more details of surface sculpture, coloration, or flagellomere shape, and are not associates of *Eucalyptus*. Our specimens of *Closterocerus chamaeleon* were reared from *Ophelimus maskelli* galls on leaves of *Eucalyptus* isolated in sealed plastic containers and lacking leaf mines. In Hansson’s (1994) key to Nearctic *Closterocerus*, *Closterocerus chamaeleon* keys to *Closterocerus ruforum* (Krausse), but these two species differ in antennal coloration especially, and in the dorsally carinate pedicel of *Closterocerus ruforum*. The pedicel in *Closterocerus chamaeleon* is rounded dorsally and lacks a carina (Fig. 2), and the scape is apically brown in *Closterocerus chamaeleon* but uniformly brownish in *Closterocerus ruforum*. Part of the 28S D2 rDNA of *Closterocerus chamaeleon* has already been sequenced (Adachi-Hagimori et al. 2011), and we are also in the process of sequencing another section of its 28S rDNA and the DNA barcoding region of its mtDNA, which will be uploaded to GenBank to facilitate identification of this species.
